# Single-Cell Sequencing: Biological Insight and Potential Clinical Implications in Pediatric Leukemia

**DOI:** 10.3390/cancers13225658

**Published:** 2021-11-12

**Authors:** Donát Alpár, Bálint Egyed, Csaba Bödör, Gábor T. Kovács

**Affiliations:** 1 HCEMM-SE Molecular Oncohematology Research Group, 1st Department of Pathology and Experimental Cancer Research, Semmelweis University, H-1085 Budapest, Hungary; alpar.donat@med.semmelweis-univ.hu (D.A.); egyed.balint@med.semmelweis-univ.hu (B.E.); bodor.csaba1@med.semmelweis-univ.hu (C.B.); 2 2nd Department of Pediatrics, Semmelweis University, H-1094 Budapest, Hungary

**Keywords:** single-cell sequencing, pediatric leukemia, cellular heterogeneity, evolutionary trajectory, targeted therapy

## Abstract

**Simple Summary:**

Leukemia is the most common type of childhood malignancy. While the clinical management of pediatric acute leukemia, especially acute lymphoblastic leukemia, underwent a remarkable improvement during the past decades, a subset of the patients still experience relapse and succumb to their disease. Emergence or repositioning of targeted therapies aided by the comprehensive characterization of single leukemic cells using advanced sequencing approaches may provide novel opportunities for therapeutic intervention in these patients. In our review, we summarize the status quo of single-cell-sequencing studies in pediatric leukemia, provide an overview of the current landscape of targeted agents applicable in this disease group, and highlight options for ways single-cell sequencing could further support the decision making related to the clinical management of pediatric leukemia.

**Abstract:**

Single-cell sequencing (SCS) provides high-resolution insight into the genomic, epigenomic, and transcriptomic landscape of oncohematological malignancies including pediatric leukemia, the most common type of childhood cancer. Besides broadening our biological understanding of cellular heterogeneity, sub-clonal architecture, and regulatory network of tumor cell populations, SCS can offer clinically relevant, detailed characterization of distinct compartments affected by leukemia and identify therapeutically exploitable vulnerabilities. In this review, we provide an overview of SCS studies focused on the high-resolution genomic and transcriptomic scrutiny of pediatric leukemia. Our aim is to investigate and summarize how different layers of single-cell omics approaches can expectedly support clinical decision making in the future. Although the clinical management of pediatric leukemia underwent a spectacular improvement during the past decades, resistant disease is a major cause of therapy failure. Currently, only a small proportion of childhood leukemia patients benefit from genomics-driven therapy, as 15–20% of them meet the indication criteria of on-label targeted agents, and their overall response rate falls in a relatively wide range (40–85%). The in-depth scrutiny of various cell populations influencing the development, progression, and treatment resistance of different disease subtypes can potentially uncover a wider range of driver mechanisms for innovative therapeutic interventions.

## 1. Introduction

Most cancers are of monoclonal origin and characterized by an unleashed proliferation of tumor cells harboring genomic, epigenomic, and transcriptomic alterations acquired in a stepwise manner or in a single catastrophic event [1,2,3,4,5]. Different clinical outcomes of patients diagnosed with the same type of cancer are largely attributed to interpatient molecular heterogeneity observed across the above mentioned molecular layers [6,7]. Indeed, recurrent aberrations identified during the past decades with karyotyping, fluorescence in situ hybridization, microarray, and other molecular methods provided a common basis for classifying patients into distinct biological and prognostic subgroups, with a subset of these cytogenetic and molecular categories also defining the most effective mode of therapeutic intervention [8,9,10].

Next-generation sequencing (NGS) has dramatically revolutionized the research and diagnostics of malignant disorders and massively expanded our knowledge of mechanisms underlying cancer evolution, including transformation, clonal expansion, adaptative selection, and treatment resistance of tumor cells [11,12]. Novel findings arising from large-scale NGS studies have led to the introduction of new disease subgroups with distinctive genomic and/or transcriptomic features and hence to the refinement of formerly established classification systems [13,14,15]. In addition, the combination of NGS with multi-region and longitudinal sampling strategies unraveled a previously underestimated level of intra-patient and intratumor heterogeneity, often coupled with complex subclonal architecture and clinically relevant temporal changes of cancer cell populations [16,17,18]. Initially, these traits and mechanisms were indirectly inferred from bulk sequencing data based on, for example, different abundance and non-ubiquitous distribution of detected genetic variants or from averaged gene expression profiles of tumor cells residing in distinct spatial localizations. While from a technical point of view, signals detected in bulk-sequencing experiments are typically more stable and less prone to variation, analysis of single cells representing the basic units of selection during cancer evolution is indispensable to the precise spatiotemporal dissection of mechanisms associated with the disease initiation as well as development, progression, and treatment resistance of various tumor cell populations [19].

Single-cell sequencing (SCS) has been providing an increasingly deep insight into the genetic composition, subclonal architecture, regulatory network, gene expression profile, and even proteome-level phenotype of a wide range of cancers [20]. The amount and density of biological information that can be generated in a single experiment by the latest large-scale SCS technologies vastly outcompete the yield of other more conventional single-cell methods, such as karyotyping, in situ hybridization, immunophenotyping, flow cytometry, or mass cytometry, commonly used for investigating blood cancers [21]. Moreover, recently emerging approaches providing multimodal NGS datasets at the single-cell level allow to investigate the interplay between different classes of biomolecules, eventually facilitating the combined analysis of cell types, cell states, development trajectories, and cellular functions in patient samples at diagnosis and upon treatment [22].

Due to the easy accessibility of non-adherent, single leukemic cells, oncohematological malignancies have commonly been pivotal in elucidating key biological mechanisms, thus fueling the progress of cancer research and facilitating the development of novel therapeutical modalities [23]. Not surprisingly, blood cancers are also among the entities most commonly analyzed in SCS studies, where generation of high-quality, single-cell suspensions is of key importance [24,25]. In line with this, leukemia samples as well as lymphoma cells circulating in the peripheral blood were the subjects in hematology-focused projects of early days [26,27], with acute myeloid leukemia (AML) being the most frequently published oncohematological entity [28]. To date, SCS datasets have been generated for a wide range of blood cancers, including chronic myeloid leukemia [29], myeloproliferative neoplasms [30,31], myelodysplastic syndrome/acute myeloid leukemia [21,27,31,32,33,34,35,36,37,38,39,40,41,42,43,44,45,46], acute lymphoblastic leukemia (ALL) [47,48,49,50,51,52,53,54,55,56,57,58], chronic lymphocytic leukemia [59,60,61], mantle cell lymphoma [61,62,63], follicular lymphoma [61,64,65,66], diffuse large B-cell lymphoma [61], multiple myeloma [26,67], Hodgkin lymphoma [68], Sezary syndrome [69], mycosis fungoides [70], Waldenström’s macroglobulinemia [25], as well as other types of B- and T-cell lymphoma [71,72,73].

Several papers have recently been published on the status quo of single-cell technologies and on their applications in blood cancer research [20,21,22,24,25,28,74,75,76]. In this review, we specifically focus on pediatric leukemia with an aim to summarize how SCS studies shed light on the pathobiology of this disease group and discuss how different layers of single-cell omics approaches can expectedly support clinical decision making in the future.

## 2. Genomic, Epigenomic, and Transcriptomic Background of Pediatric Leukemia

ALL represents 80% of all pediatric leukemia cases and comprises over 30 established or provisional genetic subtypes, with a significant subset of those being uncovered by advanced genomic and transcriptomic studies [13,14,57,77]. Pediatric ALL develops in multiple steps, with the initiating genomic lesion emerging in utero, as demonstrated in major genetic subtypes of the disease (i) by the identification of identical chromosomal rearrangements/gene fusions in monozygotic, monochorionic twins concordant for leukemia; (ii) by detecting the founder aberration in healthy co-twins of patients with ALL; and (iii) by backtracking the primary driver lesion in neonatal blood spots of patients after diagnosis [78,79]. Secondary aberrations required for the clinically manifest leukemia likely emerge postnatally and generate a branching subclonal architecture in the malignant cell population [80,81,82]. During the clinical disease course, leukemic cells with treatment-resistant potential gain selective advantage, and on average, a higher number of genetic alterations can be observed at the time of relapse as compared with diagnosis [83,84]. Recent studies reported distinct signatures of known or therapy-induced novel mutational processes leading to hypermutation and to the acquisition of relapse-driving, drug resistance-associated genomic aberrations [85,86,87,88]. SCS will expectedly aid the dissection of these processes, which tend to remain active during disease evolution regardless of the rise and fall of specific mutations, eventually conferring repeated relapse in a subset of the patients. Epigenomic features, such as methylation patterns and chromatin modifications, provide further layers of interpatient heterogeneity, rendering almost every patient with unique ALL [89]. Characteristic methylation signatures largely matching the underlying genetic background have been identified and provided basis for DNA methylation-based subtype prediction classifiers, contributing to the clarification of cytogenetically undefined ALL patient groups and facilitating the identification of novel gene fusions [90]. Some studies suggested that DNA methylation-based biomarkers are not independent predictive markers of patient outcome [91], while the combined analysis of methylation, DNA-protein interaction, and gene expression in a recent study has uncovered relapse-specific super-enhancers shared by a majority of patients [92], demonstrating the potential of integrative omic approaches. Large microarray and NGS-based transcriptomic studies identified novel subgroups of ALL patients previously not categorized by more conventional cytogenetic and molecular genetic methods. These include the Ph-like, *ETV6*-*RUNX1*-like, *KMT2A*-like, *ZNF384*-like, *DUX4*-rearranged, and *MEF2D* fusion-harboring subgroups, which have prognostic significance and/or are enriched for specific chromosomal translocations, mutations, tumor-suppressor gene deletions, kinase gene fusions, kinase pathway activation, or dysregulated lymphoid transcription factors, with some of these alterations also providing opportunities for targeted therapeutical intervention (Table 1) [13,57,93,94,95,96,97].

AML, constituting 15% of pediatric leukemia, is also a genetically heterogenous disease, and the genomic landscape observed in children is remarkably different from the repertoire and distribution of aberrations observed in adults [15]. Even within pediatric AML, the incidence of common chromosomal translocations follows an age-specific pattern with, for example, the *KMT2A* fusions vastly affecting infants, while the *RUNX1*-*RUNX1T1* and especially the *CBF* fusions being detectable in older children. Similarly, age-dependent differences are discernible in the prevalence of mutations. Characteristic alterations, such as *DNMT3*, *NPM1*, *IDH1*/*2*, *RUNX1*, and *TP53* mutations prevalent in adult de-novo AML, occur with much lower prevalence in children, while the *NRAS*, *KIT*, *KRAS*, *WT1*, *CBL*, *GATA2*, *ASXL2*, *SETD2,* and some additional genes more commonly affect children [15]. Similar to ALL, the genomic landscape of pediatric AML can significantly change with disease progression, as demonstrated by studies investigating matching diagnosis-remission-relapse trio samples [98]. DNA methylation and miRNA expression profiles revealed specific signatures that correspond to genetic subgroups or are associated with progression-free and overall survival, potentially applicable for molecular stratification of patients [15].

Juvenile myelomonocytic leukemia (JMML), accounting for around 2% of pediatric leukemia, is driven by mutations in the RAS pathway genes, with additional alterations in JAK/STAT signaling and in epigenetic modifier genes contributing to the leukemogenesis. Dissimilar to other types of pediatric leukemia, branching subclonal architecture and the development of relapse from an ancestral clone were not reported to be typical in JMML. Analysis of matching bulk diagnostic and relapse samples suggested that mutations identified at diagnosis persisted till relapse, and all early alterations emerged in a single dominant clone, with acquisition of additional aberrations following a linear fashion. In terms of prognostic significance, the number of mutations instead of their type seem to have prognostic relevance, with higher mutation counts being associated with shorter event-free and overall survival [99].

**Table 1:** Genetic subgroups as indication criteria for small-molecule targeted agents and guide for patient stratification in pediatric acute leukemia.

Genetic Subgroup	Possible Small-Molecule Targeted Agent or Risk Prediction	Refs
**ALL**		
Ph^+^ (*BCR*-*ABL1*)	TKI, Aurora kinase (alisertib)	[100]
*TCF3*-*PBX1*	PI3Ki (idelalisib), considerable response to chemotherapy	[101]
*TCF3-HLF*	therapy-resistant disease, BCL2i (venetoclax)	[102]
*KMT2A* rearrangement	FLT3i (quizartinib), DOT1L-i (pinometostat), proteasome inhibitor (bortezomib), HDACi ± DNMTi aHSCT when poor response to induction is detected	[103]
*ETV6*-*RUNX1*, *ETV6*-*RUNX1*-like, *DUX4* rearrangement (often coincide with *ERG*^del^)	Excellent prognosis, reduced intensity treatment may be considered	[57]
*MEF2D* rearrangement	HDACi (panabinostat), proteasome inhibitor (bortezomib)	[96]
*ZNF384* rearrangement	FLT3i (sunitinib)	[104]
*NT5C2* alteration	Anticipate thiopurine resistance, often associated with relapse	[105]
*CREBBP* alteration	Anticipate corticosteroid resistance	[106]
Hypodiploid	BCL2i (venetoclax), aHSCT	[107]
High hyperdiploid	Low-risk disease	[13]
iAMP21	High-risk disease	[108]
*NOTCH1* ^+^	PSEN1i (γ-secretase complex inhibition),	[109]
*IKZF1* ^plus^	in the presence of *IKZF1* alteration, FAKi potentiates other drugs’ antitumor effect High-risk disease (even with low MRD values)	[110]
*MYC* rearrangement	Resistant disease course	[111]
*NUP214*-*ABL1*	TKI	[112]
oncogene activation (eg. *TAL1*, *LYL1*, *LMO1*, *TLX1*, *TLX3*) by translocation with *TCR* genes	Varying, but substantial prognostic effect	[113]
*CDKN2A*/*CDKN2B* deletions	CDK4/CDK6i (palbociclib),	[114]
*KMT2A-ENL*	Despite KMT2A rearrangement, the prognosis is not poor in T-cell, ALL and aHSCT is generally avoidable	[115]
**Ph-like precursor B-cell ALL**		[116,117]
ABL class (*ABL1*, *ABL2*, *CSF1R*, *PDGFRA*, *PDGFRB*)	Dasatinib	
CRLF2 class (*IGH*-*CRLF2*, *P2RY8*-*CRLF2*, *CRLF2*^mut^)	USP9Xi, HDACi (givinostat)	
JAK2/EPOR class (*JAK2*, *EPOR*, *PAX5*)	JAKi (ruxolitinib)	
Ras/MAPK class (*NRAS*, *KRAS*, *PTPN11*, *NF1*)	MEKi (trametinib)	
Rare kinase fusions (*NTRK3*, *FGFR1*, *PTK2B*, *TYK2*, *DGKH*, *LYN*)	NTRKi (larotrectinib, *NTRK3*), ALKi/ROS1i (crizotinib, *NTRK3*), ponatinib (*FGFR1*), FAKi (*PTK2B*), TYK2i (*TYK2*)	
*IKZF1* alteration in Ph-like cases	Retinoid use potentiates TKIs’ effect	
**AML**		
*KMT2A* rearrangement	DOT1Li (pinometostat)	[118]
*NUP98* rearrangement	CDK6i (palbociclib), BCL2i (navitoclax) High-risk disease (need for aHSCT)	[119,120]
CBF-AML (inv(16): *CBFB*-*MYH11*, t(8;21): *RUNX1*-*RUNX1T1*)	KITi (dasatinib) Low-risk disease	[121]
*FLT3* activating mutations	FLT3i (midostaurin, crenolanib, gilteritinib, lestaurtinib, quizartinib, sorafenib)	[15]
(a) *FLT3*-ITD + *NPM1*^mut^ (usually without *DNMT3A*^mut^ in children)	The coincidence anticipates a favorable prognosis, outcomes are better than in *FLT3*-ITD^neg^ cases	
(b) *FLT3*-ITD *+ WT1*^mut^ or *NUP98- NSD1*	Usually unsuccessful induction, dismal prognosis, need for aHSCT	
*IDH1*/*IDH2* mutations (rare in children)	IDHi (enasidenib, ivosidenib)	[122]
*HIF1A*, *BRE* and *CLEC7A* expression levels	Drug resistance toward cytarabine, daunorubicin, and/or etoposide	[123]

Abbreviations: ALL, acute lymphoblastic leukemia; AML, acute myeloid leukemia; Ph, Philadelphia; TKI, tyrosine kinase inhibitor; aHSCT, allogeneic hematopoietic stem cell transplantation; MRD, minimal residual disease; ITD, internal tandem duplication.

## 3. Pathobiology Uncovered by Single-Cell Analysis

SCS studies performed in pediatric leukemia to date primarily investigated the subclonal architecture, clonal evolution, developmental state, transcriptional heterogeneity, immunophenotype, and immunomodulatory activity of leukemic blasts. The analyses focused on specific leukemia-propagating cell subsets or complete leukemic cell populations, occasionally also including the interrogation of additional immune cell compartments.

### 3.1. Pediatric Acute Lymphoblastic Leukemia

Gawad et al. analyzed the co-segregation of single nucleotide variants (SNVs), deletions, and immunoglobulin heavy chain (*IGH*) gene rearrangements in single leukemic cells of six patients with B-ALL (5/6 harboring *ETV6-RUNX1* gene fusion) using microfluidics-based targeted DNA resequencing [47]. With a thorough analytical approach, the authors quantified the allele dropout conferred by the limitations of the applied whole genome amplification method and determined a maximum level of allele dropout and a minimum number of mutations required to reconstruct the clonal structure in the analyzed diagnostic patient samples. Importantly, they demonstrated that it is not possible to resolve the cells in the samples into distinct clones based on the previously generated bulk allele frequency data alone, highlighting the importance of single-cell analysis. The study identified codominant clones in 5/6 patients, unveiled APOBEC-driven clone-specific cytosine mutagenic events that were preceded by the emergence of structural variants, and revealed clones arrested at different stages of B-cell development within the same patient. An updated version of the method, still relying on upfront bulk-sequencing data, was later described by Easton et al. [56].

Li et al. used SCS for dissecting the segregation of 14 SNVs in 56 cells isolated from the relapse sample of a patient diagnosed with Ph-like B-ALL, harboring *PAX5-NOL4L* gene fusion. They found that two *PRPS2* gene mutations, which were previously detected with bulk NGS, were present in different subclones, demonstrating convergent evolution during disease progression [85].

De Bie et al. performed microfluidics-based, single-cell targeted DNA resequencing and droplet-based, single-cell RNA sequencing in four patients with T-ALL [55]. The authors analyzed on average 24 tumor-associated aberrations per patient and identified up to four leukemic cell clones in the diagnostic samples. Gene expression profiling uncovered a very limited heterogeneity across the T-ALL cells. Selected genomic aberrations were also screened in single CD34^+^CD38^−^ multipotent progenitor cells and in bulk myeloid cell population isolated from the same patients at diagnosis and during remission. In some patients, mutations started to accumulate in the multipotent progenitor cells and were also detectable in the myeloid compartment, highlighting the importance of performing allogeneic instead of autologous stem cell transplantation in high-risk patients. Scrutiny of the acquisition order of mutations revealed early mutations mainly in genes of undetermined significance. Intermediate aberrations included T-cell receptor gene rearrangements, *CDKN2A/B* deletions, and gene fusions, while *NOTCH1* mutations proved to be late subclonal events, questioning the concept of *NOTCH1*-targeted therapies. 

In another study, *Alberti-Servera et al.* analyzed SNVs and small indels in 25 samples of eight patients with T-ALL using a droplet-based DNA resequencing approach, covering 305 genomic regions in 110 genes [52]. Samples were collected at diagnosis, during chemotherapy, or at relapse. More than two *NOTCH1* mutations acquired after the accumulation of disease initiating alterations were detected in half of the patients, with the major clone harboring one or two mutations and with other *NOTCH1* variants being present in minor subclones. Parallel evolution was also observed in the *NRAS* and *JAK1* genes. The study revealed a variable clonal response to corticosteroid treatment, demonstrated the capability of the technology to detect residual leukemic cells during treatment, and identified relapse driving clones at an early stage of the disease. 

Caron et al. performed RNA sequencing in single leukemic cells of eight patients with ALL (6 B-ALL and 2 T-ALL) in order to scrutinize intrapatient transcriptional heterogeneity at diagnosis [54]. They observed an association between gene expression heterogeneity and the predicted developmental state of the leukemic cells, which showed inverse correlation with the expression of ribosomal protein genes, especially in patients with T-ALL and high-hyperdiploid B-ALL. In patients with *ETV6/RUNX1*-positive B-ALL, gene expression heterogeneity at cellular level proved to be linked to metabolic regulation, B-cell activation, and cell proliferation. The study also tried to match transcriptionally defined cell subsets with genetic alterations and identified a couple of large deletions and gains, which were associated with specific gene expression clusters. Due to the sparsity of data, enrichment of somatic SNV alleles in the transcriptional clusters did not reach statistical significance. 

Witkowski et al. analyzed diagnostic, remission, and relapse samples of nine patients with B-ALL using droplet-based, single-cell RNA sequencing (scRNA-seq; 7 patients) and cellular indexing of transcriptomes and epitopes by sequencing (CITE-seq; 4 patients), with the latter approach allowing for the simultaneous interrogation of single-cell transcriptome and cell surface protein composition [49]. The authors demonstrated a significant remodeling of the non-malignant bone marrow microenvironment prior to chemotherapy and unveiled a non-classical monocyte subpopulation, which gained selective advantage within the myeloid compartment at diagnosis and relapse. Importantly, increased monocyte abundance at diagnosis proved to be predictive of inferior clinical outcome in patients with B-ALL. The leukemia enhanced the non-classical monocyte differentiation, while monocyte-targeted therapy via CSFR1 receptor blockade conferred depletion of monocytes and increased sensitivity of B-ALL blasts to tyrosine kinase inhibitor (TKI) therapy in vivo.

Wu et al. re-analyzed the scRNA-seq data generated by Witkowski et al. and integrated it with a large bulk RNA-seq dataset in order to further characterize cell-cell interactions in B-ALL-associated bone marrow microenvironment [50]. They identified significantly up- or downregulated ligand-receptor pairs in the autocrine network of B cells and in the paracrine network of B and myeloid cells, with a subset of altered ligand-receptor pairs being associated with clinical outcome. Building upon these findings, the authors established a score-based model that seemed to support prognostic predictions within and also beyond B-ALL.

Mehtonen et al. used scRNA-seq for investigating cell states and transcription factor activities during normal B-cell differentiation and compared those to leukemic cell profiles of six patients with *ETV6/RUNX1* B-ALL at diagnosis and during induction therapy (day 15) [58]. Their results suggested that *ETV6/RUNX1*-positive leukemic cell states are most similar to a pro-B state, differ between patients in cell cycle activity, express genes that reprogram the immune microenvironment, and that the induction therapy may modulate leukemic cells towards a pre-B state. Furthermore, the authors demonstrated the feasibility of monitoring the early treatment response using SCS and revealed elevated activity of specific transcription factors, e.g., ETS factors (ELK3, ERG, and FLI1), that could serve as targets for therapeutical intervention.

Anand et al. investigated patient-derived xenograft (PDX) models generated from diagnostic or relapse samples of pediatric patients with early T-cell progenitor (ETP) ALL or T-ALL using the Smart-Seq2 protocol allowing for full-length scRNA-seq analysis [53]. The authors also analyzed PDX and primary samples from adult patients and identified faster and slower cycling, stem-like cell populations in patients carrying *NOTCH1* mutations. Fast-cycling, stem-like cells showed high NOTCH activation, while the slow-cycling ones proved to be dependent on PI3K signaling and independent of NOTCH activation, explaining the low success rate that can be achieved by NOTCH inhibition in this entity. The study also demonstrated that cells with PI3K activity already exist at diagnosis, and the PI3K activation is independent of genetic mutations in the *PI3K*/*AKT*/*PTEN* genes, suggesting epigenetic rewiring as an underlying reason. In addition, the study shed light on the mode of immunomodulatory activity of leukemic cells, conferring CD8^+^ T-cell dysfunction via HAVCR2-LGALS9 interactions that could therapeutically be exploited in the future.

Candelli et al. analyzed a previously identified prednisone-dependent gene expression signature in 15 infants with MLL-rearranged B-ALL using plate-based and droplet-based scRNA-seq methods [124]. They demonstrated that classification of individual cells into resistant or sensitive groups, followed by the relative quantification of the two groups of cells may facilitate the prediction of an impending relapse. Characterization of treatment-resistant cells unveiled a quiescent nature with stemness features and elevated glucocorticoid response. Since the abundance of resistance signature widely correlated with clinical outcome across the patients, the authors concluded that the signature is rather generally associated with resistance to chemotherapy and likely not specific to prednisone.

### 3.2. Pediatric Acute Myeloid Leukemia

Despite the pioneering role of AML in early SCS studies, data generated from pediatric patients are still very limited. Walter et al. performed exome sequencing on five CD34^+^CD38^−^ blasts from three patients diagnosed with pediatric AML [38]. Two cells had to be excluded from the downstream analysis due to the lack of sequencing coverage across the vast majority of the targeted regions. SCS results were compared to exome and targeted amplicon sequencing data generated on bulk DNA samples. In this technical paper, the authors concluded that two-thirds of the real, non-artefact somatic alterations determined as being observable with all three methods could be detected by single-cell whole exome sequencing (WES). 

Demaree et al. simultaneously interrogated the genotype and immunophenotype of single blast cells in longitudinally collected samples of three patients with AML using DNA and Antibody sequencing (DAb-seq), with one patient representing pediatric AML [36]. By analyzing 49 DNA loci via targeted amplification and 23 protein markers with barcoded antibodies, the authors identified various proteogenomic patterns among the three patients. The pediatric patient harbored *MLL* rearrangement and relapsed at month 10 after induction and consolidation therapies. In this patient, mutually exclusive *KRAS* G13D and *FLT3* D835Y mutations were detected, with the latter one gaining selective advantage during disease progression. By investigating pathogenic blasts across all time points, immunophenotypic heterogeneity coupled with common genetic makeup as well as genetic diversity across blasts displaying shared malignant immunophenotype were observed, demonstrating that neither of the two layers of biological information would have been sufficient on their own to comprehensively characterize clinically relevant features in leukemic cells.

Louka et al. investigated hematopoietic stem/progenitor cells from three patients diagnosed with JMML using scRNA-seq and/or single-cell DNA sequencing (scDNA-seq), with one patient being analyzed by both methods [37]. Transcriptome analysis revealed heterogeneous aberrant phenotype with myeloid bias and distinct molecular signature as compared with matching normal control cells isolated from cord blood. Up-regulated genes included myeloid as well as stem cell and fetal genes, proliferation markers, erythroid differentiation-associated genes, and potential therapeutical targets, such as *MTOR* and *SLC2A1*. Single-cell genotyping demonstrated that driver aberrations can be backtracked to the hematopoietic stem cell compartment, which showed a high level of clonal dominance and contained a small number of wild-type cells. Emergence of aberrations created linear and branching clonal evolutionary patterns, with RAS-pathway mutations being detected as the earliest alterations. 

The studies briefly summarized above and listed in Table 2 demonstrate the power and versatile applicability of SCS to scrutinize pediatric leukemia blast populations as well as various immune cell subsets in the same sample at the genome, transcriptome, and proteome levels, eventually providing a highly granular profiling of intrinsic features and microenvironmental interactions that contribute to the development, progression, and treatment resistance of pediatric leukemia.

## 4. Clinical Aspects of Blast-Level Pathobiological Data

Pharmaceutical research in oncotherapy had to keep pace with the rapid advancement of data generation facilitated by the accelerated development of molecular methods during the past decade. Translation of the enormously growing data, related to acute leukemia (AL) pathobiology, to a certain patient frequently needs data-mining approaches [56,125]. Main questions for the clinical practice of modern pediatric hematology as a response to novel genomic, epigenomic, and transcriptomic findings are as follows: (i) how can we better stratify patients and optimize treatment according to risk of relapse or refractory disease course; (ii) which clonal or subclonal alterations can allow for the administration of on- or off-label small-molecule targeted agents; and (iii) how can we therapeutically benefit from the monitoring of treatment-associated dynamic changes in the genomic and transcriptomic profiles? Besides providing valuable information, single-cell analysis generates additional challenging questions, mainly related to the integration of intra-patient heterogeneity of leukemic cells into clinical decision-making systems. Importantly, this requires an *a priori* extraction of clinically relevant heterogeneity from the biological heterogeneity uncovered by advanced molecular methods.

In AL, the predisposing germline genetic material, the repertoire of abundant mutations of de novo disease and that of relapsed leukemia are significantly different; hence, the biological behavior and treatment responsiveness of AL in patients may show substantial changes over time [83,85,98]. Clonal mutations in relapsed leukemia are often traceable back to subclones prevailing during the de novo disease period. Recent literature reports clinically relevant information about the temporal dynamics of mutations associated with relapse. First, relapse-fated minor clones already exhibiting resistance to first-line chemotherapy are often present at the time of diagnosis [126]. In a study by Waanders et al., genomic variants in relapse-driver genes, such as *CREBBP*, *IKZF1,* and *NT5C2*, were rarely lost if present at diagnosis or in early therapy phase of ALL. Therefore, early detection of these alterations, which requires minimal residual disease (MRD)-level targeted screening, may predict an increased probability of treatment failure and may provide a rationale for therapy intensification or novel drug approaches before further evolution of leukemia towards relapse [86]. Ediriwickrema et al. assessed MRD in patients with relapsed AML using single-cell and bulk NGS methods. Using SCS, they identified persistent mutations in 40–50% higher number of cases, further demonstrating the potential advantage of this approach [40]. Second, putative secondary AL evolution, even in patients with lineage shift, is usually not a second independent event but represents disease recurrence arising from an ancestral pre-leukemic clone of primary leukemia [86]. From a therapeutic point of view, “real secondary AL” should be separated from “genuine relapse”. Secondary AL does not occur due to the low intensity of therapy but can be induced by mutagenic effect of overtreatment. Hence, patients in these cases face a new disease rather than a refractory leukemia course, which directs the clinician towards the application of standard frontline chemotherapy instead of following a more intensive relapse guideline. Nevertheless, allogeneic hematopoietic stem cell transplantation (aHSCT) should also be taken into account in case of “real secondary AL” to replace the predisposed leukemia stem cells [127]. Differential identification of second leukemia and relapse consists of immunoglobulin or T-cell receptor and/or fusion-gene-based clonality testing as well as cytogenetic and immunophenotypic examinations. This laborious and rather time-consuming workflow could be streamlined or at least greatly supported by properly designed SCS. Third, differential diagnosis of primary leukemia relapse and donor cell leukemia (DCL) after aHSCT may be challenging from both clinical and analytical points of view. We have previously reported a male patient with *BCR*-*ABL1*-fusion-positive ALL, whose numerical sex chromosome aberrations and loss of chromosome Y resulted from late clonal evolution caused difficulties in the interpretation of chimerism analysis at the time of relapse, after sex-mismatched aHSCT [128]. Without applying a combination of various techniques, the phenomenon could have been misdiagnosed as DCL. Since single-nucleotide polymorphism (SNP) genotyping is also feasible with SCS, this novel method could replace more conventional short tandem repeat (STR) analyses or complement sex-chromosome-based chimerism assessment after aHSCT and facilitate the detection of leukemia with or without donor cell origin.

Targeted immunotherapy is an emerging therapeutic option to achieve remission or constitute a bridge to aHSCT in childhood ALL. Currently, we are not aware of all the factors determining the clinical efficacy of boosting the anti-leukemia immune activity of own or donor immune cells. The thorough cell-level examination of tumor and immune cell clonality by SCS can identify new predictive biomarkers for immunotherapy. Rabilloud et al. used scRNA-seq to identify a relapse-driving CD19^−^ subclone allowing ALL cells to evade CD19-targeted donor chimeric antigen receptor T-cell (CAR-T) therapy in a patient [129]. In another study, response rate to bispecific antibody blinatumomab seemed to depend on the patients’ T-cell maturation state and clonality, as determined in SCS experiments using adult B-ALL samples [130]. These early results may pave the way for developing sequencing-based biomarkers associated with immunotherapy effectiveness in ALL.

Putative genomics-based stratification criteria in childhood AL expand in a rapid manner, in parallel with deeper genomic-transcriptomic characterization of real-world patient cohorts. Recent data on genetic subgroups as well as on prognostic significance of alterations and/or potentially applicable targeted therapies are summarized in Table 1. In pediatric ALL, favorable prognostic genetic events include *ETV6*-*RUNX1* fusion, *ETV6*-*RUNX1*-like expression pattern, *DUX4* rearrangement, and high hyperdiploid karyotype, while *BCR*-*ABL1* fusion, intrachromosomal amplification of chromosome 21 (iAMP21), *IKZF1*^plus^ copy number profile, and *TCF3*-*HLF* fusion are associated with inferior outcomes [131,132]. Certain alterations were found to be associated with resistance against backbone cytoreductive agents in ALL protocols (e.g., *CREBBP* aberrations predict corticosteroid resistance [106], while some studies suggested that *NT5C2* alterations may predispose to poor response to 6-mercaptopurine [105]). In pediatric AML, *NUP98* rearrangement, special activating alteration of *FLT3* (internal tandem duplication, ITD), and *WT1* mutations confer poor prognosis and drive the clinician towards aHSCT. *DNMT3A* mutations, representing early clonal events in adult AML, are associated with decreased sensitivity to anthracyclines, although they are rare in childhood [15,133]. Co-detection and relative quantification of the aforementioned tumor genetic signature in single blast cells by SCS is anticipated to provide more accurate, previously unraveled prognostic information for patient stratification as it was seen in other cancer types [134,135].

In pediatric leukemia, interrogation of recurrent genomic aberrations at the single-cell level seems especially be crucial in the light of some recent studies that have deeply scrutinized the association of clonal and subclonal alterations with clinical outcome. Jerchel et al. analyzed RAS pathway mutations in B-ALL and found that clonal but not subclonal aberrations are associated with dismal clinical outcome [136]. Antic et al. assessed the clinical relevance of subclonal alterations in eight relapse-associated genes, including *CREBBP, IKZF1, KRAS, NRAS, NT5C2, PTPN11, TP53,* and *WHSC1,* and found no correlation between these aberrations and unfavorable clinical outcome [137]. On the other hand, Barz et al. analyzed *NT5C2* mutations in a large cohort of relapsed ALL patients and observed that subclonal but not clonal mutations were associated with inferior EFS and with a higher nonresponse rate to relapse treatment. Nonetheless, in the vast majority of cases, these subclonal *NT5C2* mutations disappeared by the time of treatment failure or second relapse, indicating that mechanisms driving relapse progression have a previously underappreciated complexity and cannot just simply be attributed to mutations associated with the occurrence of the first relapse [138]. These examples clearly highlight the need for a deeper understanding of progression- and resistance-associated subclonal dynamics in pediatric leukemia, which could be greatly facilitated by SCS.

## 5. Emerging Targeted Therapies and Related Clinical Decision Making

**Figure 1:**
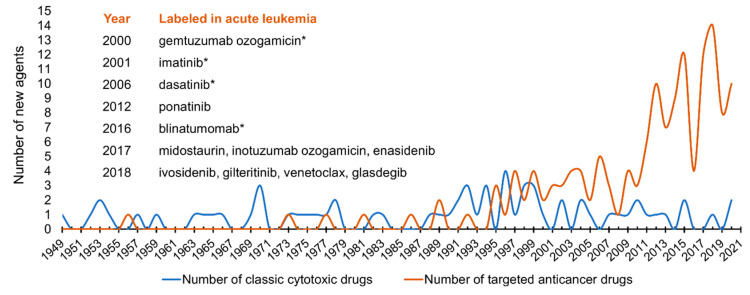
Emergence of anticancer drugs as approved by the United States Food and Drug Administration. Small-molecule targeted agents with acquired label in acute leukemia are specifically indicated. Agents with on-label approval in pediatric leukemia are marked with asterisk (*).

**Figure 2:**
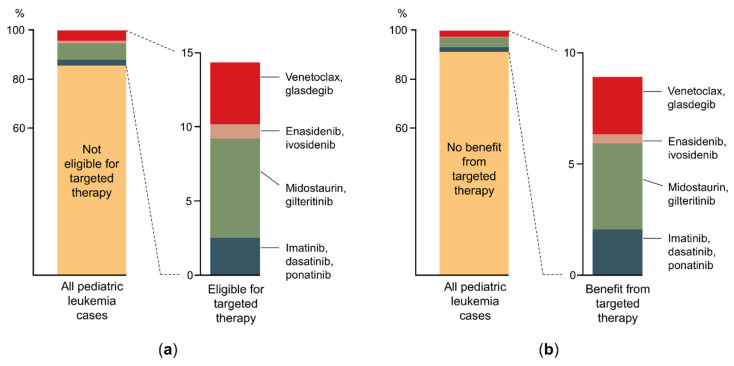
Current niche and estimated efficacy of small-molecule targeted agents in the clinical management of pediatric acute leukemia. (**a**) Proportion of patients with pediatric acute leukemia who are eligible for targeted drugs with on-label indications in acute leukemia based on genetic features. (**b**) Estimated proportion of children with acute leukemia who are expected to benefit from the currently available, on-label small-molecule targeted therapies based on average overall response rates.

Targeted molecular inhibition of cancer growth has had a previously unprecedented impact on the pharmaceutical industry: in the 2010s, the United States Food and Drug Administration (FDA) approved more small-molecule targeted agents (SMTAs) than altogether in the previous six decades (Figure 1). Development and widespread introduction of a new agent in clinical care takes several years; yet, there are more than 60 clinical trials in 2021 investigating SMTAs on the basis of fulfilled molecular genetics criteria in pediatric AL [139]. Considering the on-label indications, 12 targeted anticancer agents or combinations are approved by FDA for the treatment of AL in the United States (listed as a caption in Figure 1). Three of them are immunotherapy products, and only four of them have an on-label indication in childhood AL. We investigated what proportion of pediatric AL population would be eligible for the on- or off-label use of the nine SMTAs based on genetic features (Figure 2a). In total, only 14.4% of pediatric patients meet either of the current indication criteria. Additionally, we estimated the proportion of children with AL who could benefit from the use of these SMTAs indicated in AL based on literature data on overall response rates (ORR) to the certain agents (Figure 2b) [140,141,142,143,144,145,146]. According to our calculations, 8.9% of pediatric ALL patients could potentially benefit from SMTAs indicated in AL; however, ORR moves on a wide scale between 40 and 85%. These results suggest a currently low level of genetic data utilization in pediatric AL therapy, while the rise of this rate is reasonable to expect in the future. To date, several SMTAs are only demonstrated to be effective in preclinical models (xenografts and cell lines) of AL or labeled in other types of cancer. The application of targeted agent options listed in Table 1 relies partially on such results and not on successful clinical trials. Certainly, the indication of new SMTAs typically broadens over time; hence, those can eventually be used in a broader range of tumor types. TKIs deserve higher attention, as they constitute a backbone of *BCR*-*ABL1*^+^ pediatric ALL treatment. The introduction of TKIs brought a breakthrough in the cure of this patient group, which previously had aHSCT as the only efficient treatment option. Recently, TKI administration has also emerged in other genetic subgroups. A surprisingly rapid and durable response was observed to dasatinib in *NUP214*-*ABL1*^+^ T-cell and *NCOR1*-*LYN*-fusion-positive *BCR*-*ABL1*-like ALL patients [112,147]. Subgroups of *BCR*-*ABL1*-like ALL may constitute firm druggable entities (Table 1), but more clinical data is needed to confirm the efficacy of SMTAs in this group. In pediatric AML, several genomic-transcriptomic alterations manifest in an age-dependent manner. For example, *KMT2A* and *NUP98* rearrangements are typically prevalent in childhood, while mutations in *IDH1*, *IDH2,* and *DNMT3A* appear in adult AML [144]. Consequently, SMTAs developed for adult AML are difficult to match with pediatric treatment guidelines.

Availability and affordability of genomic profiling conferred by the spread and decreasing cost of NGS facilitated the utilization of genetic data in clinical decision making. Over the past decade, parallel progress in genomics technologies and genomics-driven drug discovery has created an opportunity to test whether a broader knowledge of genetic aberrations present in an individual’s tumor can guide treatment selection and lead to improved outcome of a certain patient. The clinical team responsible for childhood cancer care had to capitalize this opportunity and establish pipelines encompassing all steps from in-depth tumor characterization to personalized treatment suggestions. This ambition founded multidisciplinary molecular tumor boards (MTBs) composed of experts, including members from pediatric oncology/hematology, molecular pathology, cytogenetics, medical genetics, cancer biology, and bioinformatics. MTBs usually hold weekly meetings to discuss all patient related results and suggest treatment modifications or genetic counseling if a cancer-risk gene constellation has been uncovered. As examples, some highly organized pediatric MTBs are listed in Table 3. Between 2012–2018, more than 700 pediatric patients were analyzed by deep genomic characterization, e.g., WES or array comparative genomic hybridization [148,149,150,151,152,153,154]. SCS was not documented as an examination method in these studies. Approximately 15% of the included patients were referred with AL. Targetable genomic alterations were identified in 64% of them. Individualized interventions were taken in only 13% of the cases based on actionable integrative clinical sequencing findings. The most common reasons for the limited use of SMTAs was the paucity of safety and efficacy data in pediatric tumors, limiting the motivation of treating physicians to offer these treatment options to their patients. Additionally, the process required to obtain non-approved experimental drugs for pediatric patients is time consuming, and the access to these drugs is rarely granted for younger patients. The above-mentioned numbers underline the urgent need for expanded, biomarker-driven, cross-entity phase I/II combination trials for pediatric patients.

Hopefully, the precision oncology approaches will be further accelerated by SCS, which may drive the clinicians’ choice and widen the indication spectrum of SMTAs. A cell line study by McFarland et al. proposed that long-term effect of an SMTA on tumor cell viability might be predicted more precisely based on short-term (6–24 h post-treatment) transcriptional response monitoring than by multiomic characterization of the initial tumor sample [155]. In this study, transcriptional response was profiled by scRNA-seq in order to rapidly assess drug sensitivity of primary tumor cells *ex vivo*, circumventing the labor-intensive and time-consuming tasks of primary cell culturing needed to achieve sufficient cell numbers for standard viability tests. Recently, single-cell transcriptomics was also successfully used for examining the effectiveness of prednisolone in eliminating relapse-initiating clones and identifying patients with need for intensified treatment in *MLL*-rearranged infant ALL [124].

## 6. High-Resolution Cellular Characterization for Future Clinical Management

Studies focusing on the translation of NGS data acquired at single blast level to clinical decision making are in their early infancy. Most clinically oriented SCS experiments provide information on the clonal architecture of leukemia, including very minor subpopulations, and on subclonally co-occurring genetic alterations, which may modify targeted treatment strategies. As mentioned before, special relapse-fated blast subpopulations, so-called diagnosis relapse-initiating (dRI) clones, can be identified prior to chemotherapy initiation in childhood B-cell precursor ALL [126]. These subclones are both genetically and transcriptionally related to relapse and have a common ability to populate various leukemic compartments in the patient’s body. Clonal divergence of leukemic cells between the central nervous system (CNS) and bone marrow niche was suspected in a subset of patients based on previous high-throughput bulk Ig segment sequencing results by Bartram et al. [156]. Targeted sequencing by Dobson et al. corroborated these findings by unveiling genetic discordance between CNS and bone-marrow-residing blasts in 40% of examined xenograft models and providing evidence for dRI clone engraftment in the CNS [126]. 

SCS also aided an advanced understanding of developmental aspects of blast cell biology in childhood ALL [54]. In a study by Caron et al., intraindividual transcriptional clusters were identified in the majority of analyzed patients, which can indicate deregulated genes in potentially resistant subclones. However, the clinical significance of these findings needs further clarification, as it is yet unknown whether blasts at various developmental changes or with variable maturation potential have indeed fitness advantage during chemotherapy. In *ETV6*-*RUNX1*^+^ ALL, scRNA-seq revealed elevated activity of B-lineage differentiation transcription factors (TFs), also including the former leukemia genome-wide association study hit ETS domain-containing protein Elk-3 (ELK3) [58]. Leukemic TF activities persist during chemotherapy and thus provide potentially exploitable vulnerability option to overcome resistance. SMTAs targeting ETS-family TFs, like ELK3, proved to be effective in drug-resistant leukemic cells. Developmental arrest in T-cell ALL was identified by scRNA-seq as well. Consequent differential activation of pre-TCR LCK and B-cell lymphoma 2 (BCL2) signaling provide new opportunities for targeted therapy, as Gocho et al. described association of kinase inhibitor dasatinib sensitivity with pre-TCR LCK activation level [157].

SCS datasets also identified therapeutically relevant information in JMML hematopoietic stem/progenitor cells (HSPCs), where reduced clonogenicity and induced apoptosis was observed after glucose transporter 1 (GLUT1) inhibitor treatment, which seemed to be synergistic with mitogen-activated extracellular signal-regulated kinase (MEK) inhibition [37]. Even aHSCT, the only curative therapy in JMML, carries a high rate of relapse that is often difficult to recognize immediately. At a relapse following aHSCT, Louka et al. showed striking recurrence of mutant HSPCs resembling the same phenotype identified at JMML diagnosis. Interestingly albeit not surprisingly, these mutant HSPCs were present before profound molecular or clinical evidence of relapse. In an extraordinary case of a young adult with *IDH1*^+^ AML, SCS demonstrated clonal dominance gain after targeted therapy [158]. Expansion of an originally very minor *JAK2* V617F mutated cell subset (variant allele frequency at AML diagnosis: 0.6%) was observed as a response to selection pressure conferred by multiple lines of chemotherapy and isocitrate dehydrogenase 1 (IDH1) inhibitor ivosidenib treatment. Single-cell analysis greatly helped to deconvolute the clonal architecture of the patient’s myeloid malignancy, as it revealed shared *JAK2* and *IDH1* mutations in the majority of subclones. In such a case, combination therapy of SMTAs might be highly beneficial relative to Janus kinase (JAK) or IDH inhibitor monotherapy, as previously published preclinical data suggest [159].

These examples demonstrate the potential of SCS to support decision making in a clinical environment, but further translational studies are clearly required for clarifying the robustness of the method and for assessing the feasibility of its implementation in the diagnostic workflow of pediatric leukemia. Strikingly, SCS has started to emerge in currently running clinical trials with an aim to pinpoint the cell of origin of leukemogenic alterations formed in utero or to assess clonal diversity and evolution of pre-leukemic and leukemic populations with a view to correlate these findings with somatic mutation monitoring, in vitro chemotherapy resistance, MRD, and patient outcome [160,161].

## 7. Conclusions

SCS has been expanding our knowledge of the biological background of pediatric leukemia, and several studies reported novel findings with direct or indirect therapeutic relevance, potentially influencing the clinical management of patients in the future. Driven by recent technological advancements, the number of simultaneously analyzable cells and approaches supporting multimodal data generation have been permanently increasing, paving the way towards a comprehensive multiomic data landscape. The current number of papers reporting single-cell studies in pediatric leukemia is still very limited, and the widespread application of SCS methods in general faces multiple challenges posed by data sparsity, quantitative uncertainty, incomplete reference databases, relatively high cost, batch effects, and the rapid emergence of computational tools developed outside the recommended frameworks of standardized benchmarking [74]. The practical potential of SCS in large-scale research programs and clinical applications will be determined by its robustness and flexibility under non-uniform wet-lab and dry-lab conditions, which will likely require widely accepted, high-level consensus on methodological considerations [162]. From an evolutionary perspective, the fundamental unit of selection is the single cell, and in this regard, SCS enables a deep characterization of neoplasms with an ultimate resolution; however, the full potential of SCS methods can only be exploited with a more profound incorporation of evolutionary principles into the clinical decision making system of pediatric leukemia [163].

## Figures and Tables

**Table 2:** Single-cell sequencing studies on primary samples of patients with pediatric leukemia.

First Author	Year	Entity	Number of Patients ^1^	Number of Cells Analyzed	Method	Refs
Gawad et al.	2014	B-ALL	6	1479	scDNA-seq	[47]
Easton et al.	2017	ALL	1	128	scDNA-seq	[56]
Walter et al.	2017	AML	3	3	scDNA-seq (WES)	[38]
De Bie et al.	2018	T-ALL	4	1507 and 8297	scDNA-seq and scRNA-seq	[55]
Li et al.	2020	B-ALL	1	56	scDNA-seq	[85]
Caron et al.	2020	T-ALL	8	39,375	scRNA-seq	[54]
Mehtonen et al.	2020	B-ALL	6	44,746	scRNA-seq	[58]
Witkowski et al.	2020	B-ALL	9	34,407 and 42,621	scRNA-seq and CITE-seq	[49]
Wu et al. ^2^	2021	B-ALL	7	38,860	scRNA-seq	[50]
Alberti-Servera et al.	2021	T-ALL	8	108,188	scDNA-seq	[52]
Demaree et al.	2021	AML	1	14,465	DAb-seq	[36]
Candelli et al.	2021	B-ALL	15	30,909	scRNA-seq	[124]
Louka et al.	2021	JMML	3	645 and 17,547	scDNA-seq and scRNA-seq	[37]

Footnotes: ^1^ Pediatric patients are indicated. ^2^ Partial re-analysis of the dataset generated by Witkowski et al. Abbreviations: ALL, acute lymphoblastic leukemia; AML, acute myeloid leukemia; scDNAseq, single-cell DNA sequencing; scRNAseq, single-cell RNA sequencing; WES, whole exome sequencing; CITE-seq, cellular indexing of transcriptomes and epitopes by sequencing; DAb-seq, DNA and Antibody sequencing.

**Table 3:** Some examples of tumor genome profiling and pediatric molecular tumor board (MTB) initiatives in hematological or solid tumor cases.

Name of MTB (Country)	Launch Year	New Aspects, Perspectives	Leukemia Included	Refs
iCAT (United States)	2012	- Identification of translocations is needed early in the disease course when clarification of diagnosis may lead to a change in therapeutic approach	No	[154]
Peds-MiOncoSeq (United States)	2012	- Novel aberrations can be identified by comprehensive genomic profiling, which suggest new directions in translational research (e.g., this study described *ALK* fusion in rhabdomyosarcoma and *NTRK1* fusion in infantile fibrosarcoma)	Yes	[153]
PIPseq (United States)	2014	- Challenging diagnoses could be clarified (e.g., Maffucci syndrome, AMKL, gamma-delta T-cell lymphoma) - Inclusion of RNA-seq in a sequencing platform is highly advantageous because it can trustfully identify fusions which are frequently targetable	Yes	[150]
TRICEPS (Canada)	2014	- Tumor mutational burden (TMB) analysis can identify pediatric highly mutated tumors, which are candidates for immunotherap - About 10% of patients carry an underlying hereditary cancer-predisposition gene, making the identification of relevant germline variants inevitable during NGS experiments	Yes	[151]
INFORM (Germany)	2015	- The whole workflow from sample processing to target report discussed by MTB experts takes 28 days - A unique prioritization algorithm was introduced to categorize druggable and tumor biologically relevant molecular findings	Yes	[148]
LEAP (United States)	2016	- NGS panels should be part of the standard leukemia diagnostic evaluation for all pediatric patients	No	[149]
ZERO (Australia)	2017	- Invitro, high-throughput drug screening and patient-derived xenograft drug efficacy testing also helped clinical decision making	Yes	[152]

Abbreviations: INFORM, Individualized Therapy for Relapsed Malignancies in Childhood; LEAP, Leukemia Precision-based Therapy; NGS, next generation sequencing; PIPseq, Precision in Pediatric Sequencing Program; RNA-seq, ribonucleic acid sequencing; AMKL, acute megakaryoblastic leukemia; TMB, tumor mutational burden; iCAT, Individualized Cancer Therapy; ZERO, Zero Childhood Cancer.
